# Antimicrobial Potential of Biosynthesized Silver Nanoparticles by *Aaronsohnia factorovskyi* Extract

**DOI:** 10.3390/molecules26010130

**Published:** 2020-12-30

**Authors:** Fatimah Al-Otibi, Reem A. Al-Ahaidib, Raedah I. Alharbi, Rana M. Al-Otaibi, Gadah Albasher

**Affiliations:** 1 Department of Botany and Microbiology, College of Science, King Saud University, P.O. Box 22452, Riyadh 11495, Saudi Arabia; reem.bb11@gmail.com (R.A.A.-A.); raalharbi@KSU.EDU.SA (R.I.A.); ra_6n@hotmail.com (R.M.A.-O.); 2 Department of Zoology, College of Science, King Saud University, P.O. Box 22452, Riyadh 11495, Saudi Arabia; galbeshr@ksu.edu.sa

**Keywords:** *Aaronsohnia factorovskyi*, ethanolic extract, silver nanoparticles, antifungal, antibacterial, FT-IR, Zeta potential

## Abstract

The green biosynthesis of nanoparticles by plant extracts is an attractive and promising technique for medicinal applications. In the current study, we chose one of the daisy plants, *Aaronsohnia factorovskyi* (which grows in the Najd region, Saudi Arabia), to investigate its anti-microbial efficacy, in combination with silver nanoparticles. The biosynthesized nanoparticles were evaluated for antibacterial activity against *Staphylococcus aureus, Bacillus* *subtilis* (Gram-positive), *Pseudomonas aeruginosa,* and *Escherichia coli,* (Gram-negative) using the disc diffusion method, while the antifungal activity was assessed against *Fusarium oxysporum, Fusarium solani, Helminthosporium* *rostratum, and Alternaria* *alternata.* The potential phytoconstituents of the plant extracts were identified by Fourier-transform infrared spectroscopy (FT-IR) techniques, the Field emission scanning electron microscopy (FE-SEM), Chromatography/Mass Spectrometry (GC-MS) techniques, and Zeta potential analysis. The current study revealed the ability of the tested plant extract to convert silver ions to silver nanoparticles with an average diameter of 104–140 nm. Biogenic *Aaronsohnia factorovskyi*-silver nanoparticles (AF-AgNPs) showed significant antibacterial activity against *Staphylococcus aureus* with inhibition zone diameter to 19.00 ± 2.94 mm, and antifungal activity against *Fusarium solani,* which reduced the growth of fungal yarn to 1.5 mm. The innovation of the present study is that the green synthesis of NPs, which is simple, cost-effective, provides stable nano-materials, and can be an alternative for the large-scale synthesis of silver nanoparticles.

## 1. Introduction

Currently, great efforts are being made in the field of medicinal microbiology to find suitable alternatives to commonly known antibiotics, which became less effective because of the elevated bacterial resistance reported in the last decade [1]. Many studies have shown the antimicrobial activities of some medicinal plants and herbs against different bacterial and fungal species. Of these plants, the members of *Asteraceae* family, approximately 23,600 species, include different classes of sesquiterpenes that proved many applicable biological activities including the anti-inflammatory, antimicrobial, and anticancer effects, as they are important chemo-preventive agents rich in antioxidants [2,3]. *Aaronsohnia factorovskyi (A. factorovskyi*) is a member of *Asteraceae* family, which includes several medicinal plants, such as *Matricaria chamomilla* and *Helianthus annuus* (sunflower) that are characterized by their rich content of sesquiterpenes, flavonoids, and coumarins, which are active constituents of interesting therapeutic importance [4]. Recently, the flora of the Middle East has attracted the attention of several researchers and scientists to investigate their biological activities, particularly the antimicrobial and antitumor effects [5].

Silver nanoparticles (AgNPs) had proved potential bactericidal, antifungal, and anti-inflammatory activities that were suggested as a promising solution to the development of antibiotic-resistant bacteria [6]. The green synthesis of AgNPs with different biological material showed an advantage over other methods as it retains the simplicity, safety, cost-effectiveness, and stability [7]. Plant extracts have been used as mediators for the synthesis of metal ions to metal nanoparticles, such as *Eucalyptus camaldulensis, Ziziphus spina christi, Calligonum comosum,* marigold flower, *Ziziphora tenuior,* and *Azadirachta indica* [8]. The ability of these plant extracts to mediate the conversion of Ag ions to AgNPs might be due to the rich content of different biologically active compounds such as flavones, ketones, aldehydes, amides, carboxylic acids, proteins, DNA, and enzymes that mediate the reduction process of Ag ions to AgNPs [9].

The current study aimed to synthesize AgNPs from aqueous extracts of *A. factorovskyi* collected from the Najd region, Saudi Arabia. These biosynthesized molecules were investigated for their potential role as antimicrobial agents.

## 2. Results and Discussion

### 2.1. The GC-MS of A. factorovskyi Showed Highly Active Antimicrobial Components

The chemical composition of *A. factorovskyi* was phytochemically examined by GC-MS analysis; its major active constituents are listed in Table 1. The chemical structure of the resulted compounds was drawn by the free online software MolView https://molview.org/. The GC-MS analysis confirmed the presence of multiple biomolecules including phenolics and terpenoids, which are known for their antioxidant and antimicrobial activities, and are essential for the combination with nanoparticles [10].

As shown in Table 1, the highest relative intensity belonged to valeric acid (48.46%) that is commonly found in the perennial flowering plants, such as *Valeriana officinalis,* which is known for its antibacterial activities against both Gram-negative (*Escherichia coli* (*E. coli*) and *Salmonella enterica typhimurium*) and Gram-negative (*Enterococcus faecalis*, *Clostridium perfringens*, *Streptococcus pneumoniae*, and *Streptococcus suis)* bacteria, as shown by Kovanda and colleagues, in 2019 [11]. The second-highest relative intensity was that of the 1,3-benzodioxol-4-ol, a phenolic benzothiazole derivative, which approved various biological importance because of its anticancer, antimicrobial, and antioxidant activities [12]. The results showed the presence of higher content of coumarin, an aromatic heterocyclic organic compound, which is known for its antibacterial activity against both Gram-positive and Gram-negative strains, and its antifungal activity against *Aspergillus niger* and *Candida albicans*, besides it exhibited an appropriate antioxidant activity, as had been tested by the DPPH (2,2-diphenyl-1-picryl-hydrazyl-hydrate) in the radical scavenging activity assay [13]. Previous studies showed that silver nanoparticles loaded with 9-anthracenecarboxylic acid showed strong antifungal activity against *Candida albicans* [14] or antibacterial activity against human bacterial pathogens [15]. Few studies had shown the antimicrobial activity of cyanogenic glucoside, such as its antibacterial effect against *Bacillus subtilis (B. subtilis), Corynebacterium spp., E. coli,* and *Shigella dysenteriae* [16,17].

### 2.2. The Biogenic Properties of A. factorovskyi Silver Nanoparticles (AF-AgNPs)

**Figure 1:**
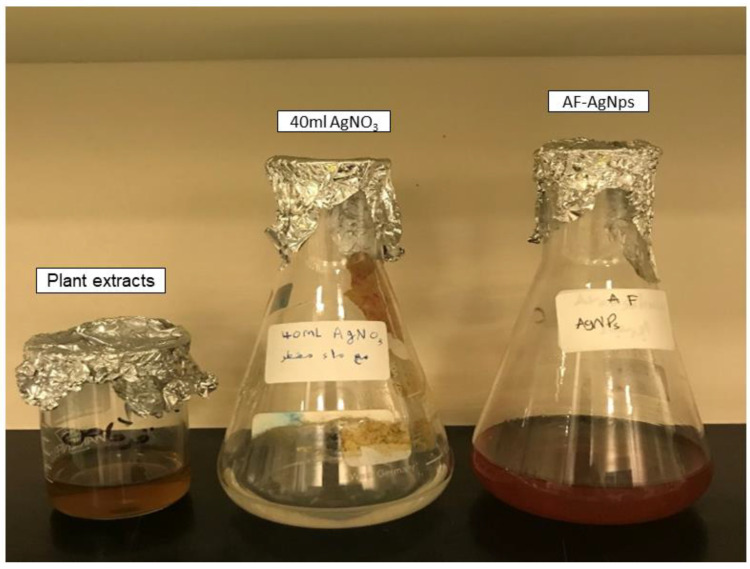
The color change in the silver nanoparticles biosynthesis.

In the current study, the constructed AF-AgNPs molecules were built up by the combination of the plant extract with AgNO_3_, which produced a colorless solution, upon subjecting to the day sunlight for 30 min, the color had changed to light yellow and turned into dark brown or reddish-brown at the end of the incubation period, as shown in Figure 1. Several studies showed similar observations: the study of the silver nanoparticles loaded by extracts from *Allophylus serratus* leaf [18] or by *Tectona grandis* seeds extract [19].

The leaves of *A. factorovskyi* were cut, rinsed, and boiled for 15 min, AgNO_3_ was diluted in distilled water to produce a clear solution that was mixed later with the *A. factorovskyi* plant extract. The mixture was subjected to sunlight for 30 min. The color changed after incubation to reddish brown.

**Figure 2:**
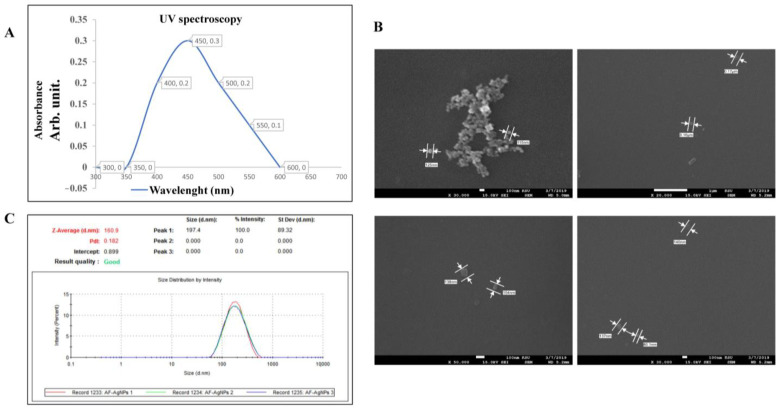
The biogenic properties of AF-AgNPs. The physical properties of the biosynthesized nanoparticles were assessed by UV-2450 Shimadzu spectrophotometer- Energy-dispersive X-ray spectroscopy (EDX) analysis and SEM. (**A**) UV visible spectra of Ag nanoparticles, (**B**) SEM results at 15 Kv, (**C**) percentage intensity of particle size distribution of biosynthesized AF-NPs. UV, ultraviolet; PdI, polydispersity index.

By the UV-2450 Shimadzu spectrophotometer, the UV visible spectra of Ag nanoparticles using the UV spectrophotometer, the presence of the expected AF-AgNPs component was confirmed, as shown in Figure 2A. A strong surface plasma resonance (SPR) peak located at 430 nm had been detected with maximum absorption OD at 0.3 arbitrary unit. This result is in agreement with another similar study conducted by Dadashpour and colleagues, in 2018, who reported the maximum absorption of the biosynthesized AgNPs, combined with *Matricaria chamomilla* at 430 nm [20].

Furthermore, we used the Scanning Electron Microscopy (SEM) to scan the presence and structural properties of the biosynthesized AF-AgNPs Figure 2B. The AF-AgNPs seem to be aggregated or separated with a uniform spherical shape. The reaction of AgNO_3_ with the extract of *A. factorovskyi* increased the diameter of the nanoparticles to approximately 104-140 nm, indicating the formation of AF-AgNPs as shown in Figure 2B. Similar studies highlighted the increase in the size of silver nanoparticles loaded with different plant extracts such as the size of silver nanoparticles from *Tectona grandis* seeds up to 27 nm [19], or 200–223 nm average diameters for the synthesized silver nanoparticles with *Cynara scolymus* leaf extracts [21]. These findings clarify that the characteristics of the biosynthesized silver nanoparticles depend on the type of the plant material extract, driven by the sunlight irradiation, besides other factors such as temperature and pH. In the current study, we used the dynamic light scattering (DLS) technique by Zeta potential analyzer to calculate the nanoparticle size, according to the intensity of the scattered light. The results shown in Figure 2C revealed that the mean diameter size of the AF-AgNPs was 197 ± 89.32 nm, with 100% intensity and Zeta average, the intensity weighted mean hydrodynamic size of the nanoparticle, was 160.9 d.nm (Z-average in nanometer).

**Figure 3:**
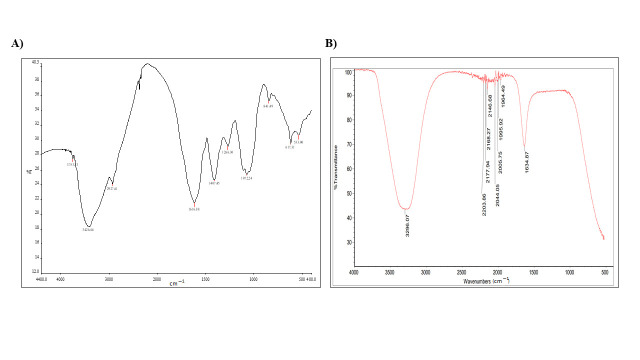
FT-IR results. The results were produced by Nicolet 6700 FT-IR Spectrometer at the range of 500–4000/cm. (**A**) *A. factorovskyi* extract, (**B**) AF-AgNPs.

FT-IR is one of the most important techniques to measure the high-resolution spectral data including the absorption or emission wavelengths of a synthesized compound [22]. The potential phytoconstituents of the plant extract, with and without silver nanoparticles, were identified by FT-IR. In the current study, the ability of the tested plant extract to react with silver ions to constitute the AF-AgNPs was revealed, as shown in Figure 3.

The FT-IR spectrum confirms the presence of various functional groups. Both the plant extract and the biosynthesized AF-AgNPs are rich in aromatic compounds, carboxylic, and alcoholic groups, alkynes and alkenes, Table 2. A similar study had suggested that the FT-IR results provided strong evidence for the presence of aromatic and nitrogenic groups that coat the silver nanoparticles to prevent their agglomeration [23]. As a member of the *Asteraceae* family, multiple studies on other plants in this family revealed similar chemical composition. In a previous study on the leaves of *Matricaria chamonbmilla* the FT-IR analysis revealed the existence of alkyl halides, alkanes, alkenes, aldehydes, and amide groups [24]. Another study revealed the presence of aromatic compounds with hydroxyl and carbonyl groups after the FT-IR analysis of *Centaurea cyanus* flower extract with another acetyl, C=C, and C=O functional groups [25].

### 2.3. The Antibacterial Activity of A. factorovskyi Extract and Silver Nanoparticles

**Figure 4:**
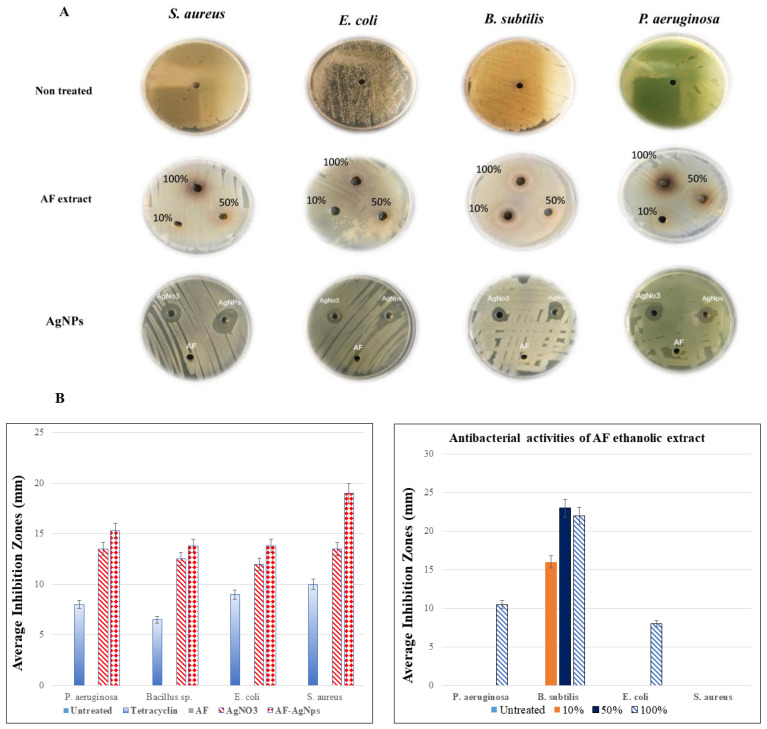
The antibacterial activity of *A. factorovskyi* extract and silver nanoparticles. The antibacterial activities of *A. factorovskyi*, Tetracycline, AF-AgNPs, and AgNO_3_ were tested against different bacterial strains, (**A**) the antibiotic diffusion results, (**B**) the average inhibition zone in (mm) for the *A. factorovskyi* ethanolic extract (right), and nanoparticles (Left).

In the current study, we tested the antibacterial activity of *A. factorovskyi* ethanolic extract against four identified bacterial strains, *Staphylococcus aureus, E. coli, B. subtilis,* and *Pseudomonas aeruginosa.* The antibacterial activity was tested against different concentrations of *A. factorovskyi* using the Kirby-Bauer disc diffusion method [26], and calculating of the average inhibition zone in millimeters by measuring the distance from the center of the disc to the borders of the area at which the growth is inhibited [27]. As shown in Figure 4, all bacterial strains were sensitive for the treatment with tetracycline. *A. factorovskyi* extract showed a strong inhibitory effect against *B. subtilis* at all concentrations, while only the 100% solution showed some inhibitory effects against *P. aeruginosa* and *E. coli* without any observed inhibition against *S. aureus*, Table 3.

Unlike *A. factorovskyi,* the AF-AgNPs showed stronger inhibition against all bacterial strains, particularly, *S. aureus* with mean average inhibition of 19.00 ± 2.94 mm, despite the fact that the water extract did not show any efficacy in the inhibition, as shown in Table 4. Noticeably, the treatment with Silver Nitrate (AgNO_3_) alone resulted in stronger inhibitory effect against all strains, suggesting that the antibacterial activity of AF-AgNPs is modulated mainly by the chemical composition of the nanoparticle and that the combination with *A. factorovskyi* will enhance and induce more inhibitory impact.

Several studies had suggested and proved the antibacterial activity of AgNO_3_ and silver nanoparticles. AgNPs showed adequate antimicrobial activity against *E. coli, Klebsiella oxytoca,* and *C. albicans* where the diameters of inhibition zones were about 10 to 23 mm; and the minimum inhibitory concentration (MIC) of AgNPs was 15 μg/mL against *E. coli* [28]. Another study showed that both AgNPs and AgNO_3_ at 20 µg/mL possessed an antibacterial effect against *Klebsiella pneumoniae, Salmonella typhi*, and *Vibrio cholerae* with inhibition zone diameters of 9, 8, and 13 mm, respectively [29]. Several studies suggested that the spherical shape, concentration, and type of silver nanoparticle might play a key role in the bacterial inhibition, which is absorbed inside the bacterial cells to interact with the bacterial DNA and proteins causing their damage [30], which further resulted in more oxidative stress through the generation of Reactive Oxygen Species (ROS) [31]. ROS accumulates into the bacterial mitochondrial membrane resulting in dysfunctional mitochondria and, hence, inhibit the bacterial growth [32]. It was reported that the antibacterial activity of AgNPs is greater against gram-negative bacteria because of their interaction with the lipopolysaccharide and thick peptidoglycan layers of the bacterial membrane and cell wall, causing its disruption [33].

In the current study, the antibacterial activity of the aqueous extracts of *A. factorovskyi* was limited and unclear compared to the ethanolic extract. The antibacterial activity had been studied previously for different types of herbs and medicinal plants organic extracts compared to water extracts. The ethanolic extract of *Rosmarinus officinalis* showed a significant antibacterial effect against *Bacillus cereus, S. aureus, E.* coli, *Salmonella enteritidis, Vibrio parahaemolyticus,* and *P. aeruginosa* where the water extract was only effective against *Bacillus cereus* [34]. Previous studies showed that the methanolic and ethanolic extracts of *Matricaria pubescens*, unlike water extract, induced a large inhibition zone against *E. coli* and Methicillin-resistant *S. aureus* with 50% growth inhibition [35], and *Acinetobacter baumannii* [36] due to the higher contents of phenolic compounds and flavonoids in these extracts. Thus, the current study suggested a potential antibacterial impact of the ethanolic extract of *A. factorovskyi* aerial parts.

### 2.4. The Antifungal Activity of A. factorovskyi Extracts and Silver Nanoparticles

**Figure 5:**
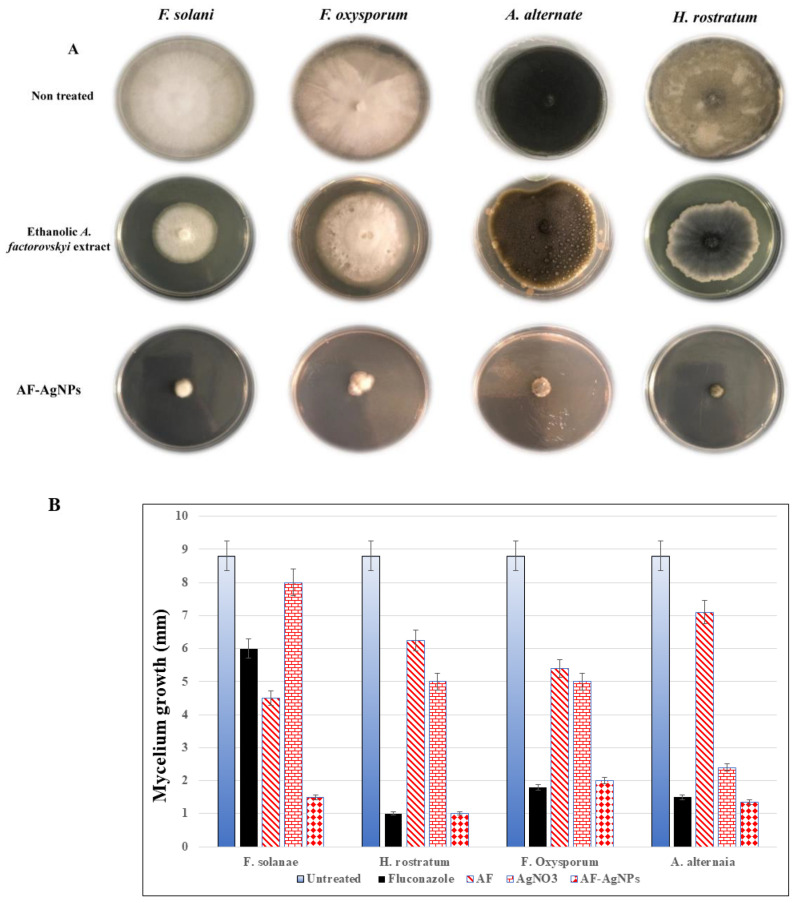
The antifungal activity of *A. factorovskyi* ethanolic extract and silver nanoparticles. The antifungal activities of *A. factorovskyi*, and AF-AgNPs, were tested against four fungal species. (**A**) The species were cultured on potato dextrose agar Petri dishes and treated with either *A. factorovskyi* ethanolic extract or AF-AgNPs. (**B**) The mycelium growth inhibition in (mm) for the fungal species treated with either *A. factorovskyi* ethanolic extract or the biosynthesized silver nanoparticles.

As shown in Figure 5, all fungi were sensitive for the treatment with Fluconazole. The effect of *A. factorovskyi* ethanolic extract has been studied against *Fusarium solani (F. solani), Fusarium oxysporum, Helminthosporium rostratum,* and *Alternaria alternata.* The results showed mild mycelium growth inhibition of *F. oxysporum* and *F. solani* by 39% and 49%, respectively compared to the control, however, the inhibitory effect was weaker against *H. rostratum,* and *A. alternata* by 29% and 19% respectively (Table 5). The treatment with AgNO_3_ induces some growth inhibition effect, despite it was not significant except for *F. oxysporum*. The treatment with AF-AgNPs resulted in significantly stronger inhibition (*p < 0.05*) with 83%, 88%, 85%, and 77% growth inhibition against *F. solani, H. rostratum, F. oxysporum,* and *A. alternata,* respectively (Figure 5). These results indicated a robust antifungal property of AF-AgNPs.

Several previous studies had reported the antifungal activities of silver nanoparticles. AgNPs showed high antifungal activity against *Rhizoctonia solani* anastomosis groups (AGs) by 73.60% mycelium growth inhibition [37]. Another study reported the antifungal property of AgNPs synthesized by *Ligustrum lucidum* leaf extract that induced 50% inhibition of *Setosphaeria turcica* at the concentration of 170.20 μg/mL [38]. In another study, the AgNPs exhibited strong antifungal activity against *Trichosporon asahii* with MIC of 0.5 μg/mL, which caused serious cell wall damage, mitochondrial membrane disruption, and other cellular changes [39]. Several studies suggested the antifungal effect of AgNPs was due to its effect on the membrane-bound enzymes, such as those included in the respiratory chain [40] or by the destruction of the cellular membrane integrity [41]. Other nanoparticles, such as ferric oxide (Fe_2_O_3_) nanoparticles, were reported to have a stronger antifungal activity against the *Cladosporium herbarium,* which was marked by ROS generation [42]. A previous study showed that the ethanolic extract of *Matricaria chamomilla* L. has a good inhibition effect on the growth of *Candida albicans* and *Candida tropicalis* because of the lipophilic essential oils rich in flavonoid and apigenin, which are known for their antifungal properties [43]. It can be concluded that AgNPs constitute an effective antimicrobial agent against common pathogenic microorganisms.

## 3. Materials and Method

### 3.1. Chemicals and Reagents

Several compounds have been used for the preparation and construction of *A. factorovskyi* extract and nanoparticles. The 99.9% Ethanol and AgNO_3_ were purchased from Sigma-Aldrich (Sigma-Aldrich Chemie GmbH, Taufkirchen, Germany). The Mueller Hinton Agar, Potato Dextrose Agar, Fluconazole, and the antimicrobial susceptibility disks (Tetracycline) were purchased from Thermo Fisher Scientific (Thermo Fisher Scientific, Waltham, MA, USA).

### 3.2. Plant Material Collection, Classification, and Preparation of Ethanolic Extract

The plant materials were collected in February 2019 from different spots in Riyadh, Saudi Arabia. The *A. factorovskyi* plants were identified and authenticated by Professor Najat Abdul-Wahab Bukhari, a taxonomist from the department of Botany and Microbiology, Faculty of Science, King Saud University, Riyadh, Saudi Arabia. In the current study, we used the aerial parts of *A. factorovskyi,* which consist of green rounded stems, divided into linear lobes with lower feathery leaves and upper yellow “button” flowers.

The ethanolic extract of *A. factorovskyi* was prepared, as it has been described before [3]. Briefly, the fresh plant parts were cut into small pieces, boiled for 15 min, shade dried at an ambient temperature of about 25–30 °C, and then powdered by vigorous grinding. To remove the large undesirable parts, a 4% solution of the plant powder was soaked in distilled water for 15 min, filtered through Whatman filter paper, and followed by air drying. The dried powder was weighed and stored at 4 °C for further experiments. On the experimental day, the powder was dissolved in absolute ethanol according to the required concentrations.

The chemical composition of the prepared samples was confirmed by the Gas Chromatography/Mass Spectrometry technique (GC-MS) using the aqueous extract of *A. factorovskyi*. The capillary-gas chromatography /mass spectrometry, GC-2010 (SHIMADZU corp., Kyoto, Japan) supplied with 30 m Rtx-5MS capillary column of 0.25 mm in diameter and film thickness of 0.25 μm with helium, as a carrier gas, and a maximum temperature of 280 °C. The isolated compounds were identified by the commercial libraries of the The National Institute of Standards and Technology (NIST) research library [44] https://www.nist.gov/nist-research-library.

### 3.3. Microorganisms

In the current study, we used four strains of fungi, *F. solani, F. oxysporum, H. rostratum,* and *A. alternata*. Besides, another four strains of bacteria *S. aureus*, *B. subtilis.* (Gram-positive), *P. aeruginosa,* and *E. coli*. The strains of fungi were identified, isolated, and classified by either the Department of Plant Protection, College of Food and Agricultural Sciences, King Saud University, or by the National Center for Research on Agriculture and Livestock, located in Riyadh, Saudi Arabia. The strains of bacteria were kindly provided by King Khaled University Hospital.

### 3.4. Preparation and Biogenic Characterization of AF-AgNPs

AF-AgNPs were prepared as shown by Mladenova and colleagues in 2018 [45]. Briefly, an amount of 85 mg of silver nitrate (AgNO_3_) was dissolved in distilled water with vigorous stirring using the BOECO Magnetic Stirrer MMS 3000 (BOECO co., Hamburg, Germany) at room temperature. Then, 0.5 mL of 4% of the ethanolic plant extract was added wisely to the AgNO_3_ particles, from the previous step, and the volume was adjusted to 100 mL with distilled water. Later, the prepared samples were subjected to the sunlight for 30 min, when the mixture color turned to an intense yellowish-brown. The biogenic characterization of the prepared nanoparticles was assessed by UV spectroscopy, DLS, FE-SEM, FT-IR, and GC-MS.

The reduction of pure Ag^+^ ions was assessed by UV-2450 double-beam UV-visible spectrophotometer (Shimadzu, Tokyo, Japan) at the wavelength range from 200 to 800 nm following the manufacturer’s recommendations, as shown before [46].

The extremal characterizes and morphology of the AF-AgNPs were distinguished by the FE-SEM technique using the JSM-7500F Field Emission Scanning Electron Microscope (JEOL, Peabody, MA, USA). Briefly, 8 μL of AF-AgNPs suspensions were located in 200 mesh grids with a carbon support film (Agar Scientific, London, UK), rinsed with ethanol, air-dried, fixed on the appropriate SEM holder where images were captured at an accelerating voltage of 30 kV, according to the manufacturer instructions.

DLS technique was conducted by Zeta potential analyzer Zeta sizer (Malvern Panalytical, Malvern, UK) to analyze the nanoparticles mobility and charge (Zeta potential) by Electrophoretic Light Scattering (ELS), according to the manufacturer instructions.

The Fourier-Transform Infrared Spectroscopy (FT-IR.) was used to evaluate the infrared (IR) absorption and emission spectra of the biomolecules content in the prepared samples by Nicolet 6700 FT-IR Spectrometer (Waltham, MA, USA) at the range of 500–4000/cm.

### 3.5. The Antibacterial Activity Evaluation

The antibacterial activity was evaluated by the Kirby-Bauer disc diffusion method as described before [26]. Briefly, the strains were cultured separately in the Mueller-Hinton Agar plates for 24 h, and the growth efficiency was evaluated by observation of the inhibition areas against antibiotic discs. On the next day, two colonies were picked and transferred to a tube of 10 mL distilled water, mixed thoroughly, and spread on new Mueller-Hinton Agar plates by a sterile cotton swab (2.5 × 10^5^ colony-forming unit (CFU)/mL). In each plate, three 4 mm diameter adequately spaced holes were made in the culture agar surface by a metal rod and 0.2 mL of either different concentration of the plant ethanolic extract, AF-AgNPs, or the control (distilled water) were loaded under aseptic conditions and incubated for 18–24 h hours at 37 °C. In parallel, the bacterial cultures were incubated with Tetracycline antibiotic discs (30 µg) to act as a positive control. The inhibition rate was evaluated by measuring the distance between the hole and the nearest growth area in mm. The minimal inhibitory concentration (MIC) of the extract was calculated using serial dilution concentrations (50, 25, 12.5, 6.25, and 3.13 mg ⁄ml) as described before [47].

### 3.6. Determination of Antifungal Activity

The antifungal activity was performed with little modifications from Andleeba and colleagues in 2020 [48] and Kim and colleagues in 2012 [41]. Briefly, either the AF or AF-AgNPs was mixed with potato dextrose agar (PDA) media at the mean concentrations, added to Petri dishes, and incubated at room temperature for 48 h. After the incubation period, 8 mm diameter agar plugs with the fungi strains were introduced to the center of each Petri dish and re-incubated at 28 ± 2°C. In parallel, the fungi were incubated with Fluconazole (32–320 μM) to act as a positive control. On the seventh day of incubation, the mycelial growth inhibition was determined by measuring the colony diameter of the cultured fungi.

### 3.7. Statistical Analysis

All the experiments were performed in triplicates. One-way ANOVA in SPSS Statistics was used to evaluate the significance levels of results at *p < 0.05* by IBM SPSS Statistics 22.0.

## 4. Conclusions

The biogenic AgNPs showed significant antibacterial and antifungal activities against all studied species particularly, *S. aureus* and *F. solani*. The innovation of the present study is that the green synthesis of NPs, which is simple and cost effective, provides stable nano-materials and can be an alternative for the large-scale synthesis of silver nanoparticles. More investigations might be conducted against other species.

## Figures and Tables

**Table 1:** GC-MS analysis results of *A. factorovskyi* ethanolic extract.

Phenolic Compound	Formula	Chemical Structure	Molecular Weight	MS Fragments (*m/z*)	Relative Intensities%
**Cyanogenic glycosides** **(2’-Epimer)**	C_16_H_19_NO_8_	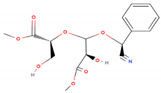	353.327	353.00	13.78
**1,3-benzodioxol-4-ol**	C_7_H_6_O_3_	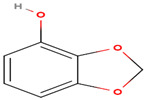	138.122	138.00	43.51
**Valeric acid**	C_5_H_10_O_2_	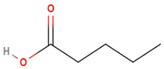	102.133	102.00	48.46
**9-Anthracenecarboxylic acid**	C_15_H_10_O_2_	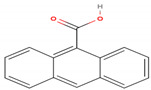	222.243	222.00	13.97
**Coumarin**	C_9_H_6_O_2_	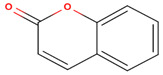	146.145	146.00	36.22

**Table 2:** The functional group analysis by FT-IR.

Tested Material	Absorption (cm^−1^)	Appearance	Group	Compound Class
AF extract	3753	Medium, sharp	O-H stretching	Alcohol
	3426	Strong, broad	O-H stretching	Alcohol
	2927	Weak, broad	O-H stretching	Alcohol
	1616	Strong	C=C stretching	α, β-unsaturated ketone
	1407	Medium	O-H bending	Carboxylic acid
	1266	Strong	C-N stretching	Aromatic amine
	1072			None
	841	Strong	C-Cl stretching	Halo compound
	617	Strong	C-Br stretching	Halo compound
	533	Strong	C-I stretching	Halo compound
AF-AgNPs	3269	Strong, broad	O-H stretching	Carboxylic acid
	2203	Weak	CΞC stretching	Alkyne
	2177, 2168	Strong	S-CΞN stretching	Thiocyanate
	2146	Weak	CΞC stretching	Alkyne
	1995, 1964	Weak	C-H bending	Aromatic compound
	2044, 2005	Strong	N=C=S stretching	Isothiocyanate
	1634	Medium	C=C stretching	Alkene

**Table 3:** Screening of the minimal inhibitory concentration (MIC) of *A. factorovskyi* ethanolic extract measuring the inhibition zone (mm) for bacterial strains.

*A. factorovskyi* Concentration of the Ethanolic Extract	*S. aureus*	*E. coli*	*B. subtilis*	*P. aeruginosa*
10%	-	-	16	-
50%	-	-	23	-
100%	-	8	22	10.5

AF; *A. factorovskyi*, -; no inhibition was noticed.

**Table 4:** The antibacterial activities of *A. factorovskyi* aqueous extract and AF-AgNPs by measuring the average of inhibition zone (mm).

Treatment	*S. aureus*	*E. coli*	*B. subtilis*	*P. aeruginosa*
Untreated	-	-	-	-
Tetracycline	8.00 ± 0.98	6.50 ± 0.15	9.00 ± 1.90	10 ± 0.66
*A. factorovskyi*	-	-	-	-
AgNO_3_	14.17 ± 0.76	12.00 ± 0.00	12.50 ± 1.50	13.50 ± 0.50
AF-AgNPs	19.00 ± 2.94	13.83 ± 0.85	13.83 ± 1.43	15.33 ± 0.47

AF; *A. factorovskyi*, AF-AgNPs; *A. factorovskyi* Silver nanoparticles, -; no inhibition was noticed.

**Table 5:** The antifungal activities of *A. factorovskyi* ethanolic extract and AF-AgNPs by measuring the average mycelium growth (mm).

Treatment	*F. solani*	*H.* *rostratum*	*A. alternata*	*F. oxysporum*
Untreated	8.80 ± 0.00	8.80 ± 0.00	8.80 ± 0.00	8.80 ± 0.00
Fluconazole	6.00 ± 0.01	1.00 ± 0.50 *	1.80 ± 0.10 *	1.50 ± 0.83 *
*A. factorovskyi*	4.50 ± 0.00	6.25 ± 0.00	7.10 ± 0.10	5.40 ± 0.00
AgNO_3_	8.00 ± 0.01	5.00 ± 0.09	5.00 ± 0.22	2.40 ± 0.08 *
AF-AgNPs	1.50 ± 0.00 *	1.00 ± 0.00 *	1.35 ± 0.15 *	2.00 ± 0.00 *

AF; A. factorovskyi, AF-AgNPs; A. factorovskyi silver nanoparticles, * p-value < 0.05.

## Data Availability

The data presented in this study are available on request from the corresponding author. The raw data are not publicly available due to large files volume produced by different techniques.
